# Draft Genome Sequence of Thauera sp. Strain SWB20, Isolated from a Singapore Wastewater Treatment Facility Using Gel Microdroplets

**DOI:** 10.1128/genomeA.00132-15

**Published:** 2015-03-19

**Authors:** Armand E. K. Dichosa, Karen W. Davenport, Po-E Li, Sanaa A. Ahmed, Hajnalka Daligault, Cheryl D. Gleasner, Yuliya Kunde, Kim McMurry, Chien-Chi Lo, Krista G. Reitenga, Ashlynn R. Daughton, Xiaohong Shen, Seth Frietze, Dongping Wang, Shannon L. Johnson, Daniela I. Drautz-Moses, Stephan Schuster, Patrick S. Chain, Cliff Han

**Affiliations:** a Bioscience Division, Los Alamos National Laboratory, Los Alamos, New Mexico, USA; b Singapore Centre on Environmental Life Sciences Engineering, Nanyang Technological University, Singapore

## Abstract

We report here the genome sequence of Thauera sp. strain SWB20, isolated from a Singaporean wastewater treatment facility using gel microdroplets (GMDs) and single-cell genomics (SCG). This approach provided a single clonal microcolony that was sufficient to obtain a 4.9-Mbp genome assembly of an ecologically relevant Thauera species.

## GENOME ANNOUNCEMENT

Species of the betaproteobacterial Thauera genus have been characterized as being able to aerobically (1–3) or anaerobically (4–7) degrade aromatic compounds under denitrifying conditions, as well as to oxidize organic acids and alcohols (8). Due to the potential of Thauera species in bioremediation applications and for their ability to produce exopolysaccharides (9), much interest has been focused on assembling and annotating the genomes of these ecologically relevant bacterial species. Interestingly, all the currently available Thauera sp. genomes were obtained from isolates from sludge/wastewater treatment facilities (9, 10).

Recently, our team utilized the combined technologies of gel microdroplets (GMDs) and single-cell genomics (SCG) to obtain several near-complete genomes of novel bacterial species inhabiting the human oral and gut microbiomes (11, 12). We applied this approach to coculture a complex sewage wastewater microbial community to attempt the recovery of as many diverse bacterial representatives as possible. We flow-sorted 176 GMDs and then subjected each to whole-genome amplification and bacterial 16S rRNA phylotyping (11–14). Of the 33 recovered bacterial genera (data not presented), one GMD was phylotyped as Thauera (named SWB20), having 99.9% 16S rRNA gene sequence identity (1,409 bp) to Thauera sp. MZ1T strain MZ1 (GenBank accession no. NR_074711.1). Due to its ecological significance, the amplified genome products of SWB20 were used to create libraries for the Illumina MiSeq (Illumina, Inc., San Diego, CA) and PacBio (Pacific Biosciences, Menlo Park, CA) sequencing platforms. The Illumina genome assemblies included both IDBA-UD version 1.1.0 (15) and Velvet version 1.2.08 (16) for 300× coverage. The PacBio genome assemblies utilized HGAP version 2.1.1 (17) for 159× coverage. The hybrid assemblies yielded 21 contigs (*N*_50_, 343,694; *N*_90_, 154,439; maximum, 1,000,345; minimum, 14,734) for a near-complete 4,927,396-bp genome with 66.2% G+C content. Only contigs >14,000 bp from the final assembly were used for the subsequent annotations (18), which showed 4,421 protein-encoding genes (PEGs), 11 rRNAs (three 16S rRNAs, four 23S rRNAs, and four 5S rRNAs), and 61 tRNAs.

We performed genomic phylogeny and single-nucleotide polymorphism (SNP) comparison analyses (our unpublished data) of SWB20 against the previously published Thauera genomes: *T. aminoaromatica* strain MZ1T (9, 19) (also known as MZ1T; GenBank accession no. NC_011662.2), Thauera sp. strain 27 (accession no. AMXB01000000), Thauera sp. strain 28 (accession no. AMXA01000000), *T. linaloolentis* 47Lol (accession no. AMXE01000000), Thauera sp. strain 63 (accession no. AMXC01000000), and *T. aminoaromatica* S2 (also known as S2; accession no. AMXD01000000) (10). We found that SWB20 clusters with MZ1T and S2, while MZ1T and S2 are more closely related (data not presented). Our SNP analysis yielded a core genome size of 1,064,233 bp across all seven Thauera genomes. SWB20 had 0.66% and 0.58% SNP composition with MZ1T and S2, respectively, while S2 had 0.41% SNP composition compared to that of MZ1T, thereby supporting the close phylogenetic topography of these three Thauera strains. Both the close phylogeny and very low SNP differences compared to MZ1T and S2 suggest that SWB20 may be a unique *T. aminoaromatica* strain. Compared to the remaining Thauera sp. genomes, SWB20 averaged ~10% SNP composition (data not shown).

### Nucleotide sequence accession numbers.

The draft genome sequence of Thauera sp. strain SWB20 has been deposited as a whole-genome shotgun project at DDBJ/EMBL/GenBank under the accession no. JTDM00000000 (BioProject ID PRJNA267225). The version described in this paper is version JTDM01000000.
